# Factors Affecting Occupational Exposure to Needlestick and Sharps Injuries among Dentists in Taiwan: A Nationwide Survey

**DOI:** 10.1371/journal.pone.0034911

**Published:** 2012-04-03

**Authors:** Hsin-Chung Cheng, Chen-Yi Su, Amy Ming-Fang Yen, Chiung-Fang Huang

**Affiliations:** 1 School of Dentistry, College of Oral Medicine, Taipei Medical University, Taipei, Taiwan; 2 Department of Dentistry, Taipei Medical University Hospital, Taipei, Taiwan; 3 Graduate Institute of Life Sciences, National Defense Medical Center, Taipei, Taiwan; 4 School of Oral Hygiene, College of Oral Medicine, Taipei Medical University, Taipei, Taiwan; 5 School of Dental Technology, College of Oral Medicine, Taipei Medical University, Taipei, Taiwan; Vanderbilt University, United States of America

## Abstract

**Background:**

Although the risks of needlestick and sharps injuries (NSIs) for dentists are well recognized, most papers published only described the frequency of occupational exposure to NSIs. Less has been reported assessing factors contributing to exposure to NSIs. The purpose of this study was to update the epidemiology of NSIs among dentists in Taiwan and identify factors affecting NSIs in order to find preventive strategies.

**Methodology/Principal Findings:**

A nationwide survey was conducted in dentists at 60 hospitals and 340 clinics in Taiwan. The survey included questions about factors supposedly affecting exposure to NSIs, such as dentist and facility characteristics, knowledge and attitudes about infectious diseases, and practices related to infection control. Univariate and multivariate logistic regression analyses were conducted to determine the association between risk factors and exposure to NSIs. In total, 434 (74.8%) of 580 dentists returned the survey questionnaires, and 100 (23.0%) reported that they had experienced more than one NSI per week. Our data showed that the risk of occupational NSIs is similarly heightened by an older age (odds ratio [OR], 3.18; 95% confidence interval [CI], 1.62–6.25), more years in practice (OR, 2.57; 95% CI, 1.41–4.69), working in clinics (OR, 1.73; 95% CI, 1.08–2.77), exhibiting less compliance with infection-control procedures (OR, 1.82; 95% CI, 1.04–3.18), having insufficient knowledge of blood-borne pathogens (OR, 1.67; 95% CI, 1.04–2.67), and being more worried about being infected by blood-borne pathogens (OR, 1.82; 95% CI, 1.05–3.13).

**Conclusions/Significance:**

High rates of NSIs and low compliance with infection-control procedures highly contribute to the chance of acquiring a blood-borne pathogen infection and threaten occupational safety. This study reveals the possible affecting factors and helps in designing prevention strategies for occupational exposure to NSIs.

## Introduction

Needlestick and sharps injuries (NSIs) present the greatest occupational risk for transmission of blood-borne pathogens such as hepatitis B virus (HBV), hepatitis C virus (HCV), and human immunodeficiency virus (HIV). It is estimated that more than 600,000 NSIs occur annually in the United States, and almost half of these events are unreported [1]. The occurrence of NSIs in Taiwan is reported to be 1.3 per person per year and is significantly higher than in other Asian countries [2]–[4]. These injuries may cause potentially fatal infections with blood-borne pathogens and are a serious occupational safety concern for healthcare workers. The risk of infection due to NSIs ranges from as high as 40% for HBV and 3%∼10% for HCV, to as low as 0.2%∼0.5% for HIV [5]–[8]. In Taiwan, healthcare workers face even greater risks because HBV is highly endemic with 15%∼20% of the general population having the hepatitis B surface antigen (HBsAg), and the number of HIV cases is also increasing [9]–[14]. However, research on potential factors in Taiwan associated with NSIs is limited.

Dentists are known to be a high-risk group for exposure to NSIs [15], [16], and most dentists experience at least one NSI during their profession life [17]. There are several reports of occupationally acquired HCV and HIV infection in healthcare workers following NSIs [18], [19], and reports of HIV transmission to patients from dentists were published during the last two decades [20], [21]. This has raised concerns about compliance with infection-control procedures, which are recommended to protect dentists against blood-borne infections [22], [23]. Although the risk of cross-infection for dentists is well recognized, most papers published only described the frequency of occupational exposure to blood-borne pathogens [17], [24], [25]. Less has been reported assessing the association between risk factors and NSIs [16], [17]. Much more needs to be learned about factors related to exposure to NSIs so that preventive efforts can be made. The purposes of this study were to update the epidemiology of NSIs among dentists in Taiwan and identify factors contributing to NSIs in order to find preventive strategies.

## Methods

### Setting

We used a multistage proportional stratified sampling method to obtain a nationally representative sample. According to government urbanization index, all 359 townships in Taiwan were divided into 2 central cities, 3 provincial cities and 16 counties. We defined our research areas as 2 central cities, 3 provincial cities and 8 counties, which were randomly selected from 16 counties. Then, a total of 60 hospitals and 340 clinics were randomly selected from the 13 research areas when a random number generated by a random number generator was less than the probability proportional to size sampling method. The multistage stratified sampling scheme has been used for several published studies [26], [27].

**Table 1:** Demographic and Practice Characteristics (*n* = 434).

Characteristic	No. (%)
Gender	
Male	317 (74.6)
Female	108 (25.4)
Age (years)	
<31	111 (25.7)
31∼40	105 (24.3)
41∼50	127 (29.4)
>50	89 (20.6)
Length of practice as a dentist (years)	
<6	120 (28.2)
6∼10	70 (16.4)
11∼15	45 (10.6)
>15	191 (44.8)
Number of patients treated per day	
<11	164 (38.1)
11∼20	201 (46.7)
21∼30	54 (12.6)
>30	11 (2.6)
Information source for dental infection control	
Newspaper or internet	74 (17.1)
Journals or textbooks	183 (42.2)
Seminars or conferences	177 (40.8)
Practicing in a setting that provides a postgraduate year for dentists	
Yes	154 (37.7)
No	255 (62.3)
Level of practice setting	
Hospital	187 (43.1)
Clinic	247 (56.9)

### Data collection

The survey included questions about factors that supposedly affect exposure to NSIs, such as dentist and facility characteristics, knowledge and attitudes about infectious diseases, and practices related to infection control. The compliance to infection control was measured from eight infection control procedures in the survey. The age of participates was classified into four categories: <31, 31–40, 41–50, and >50 years. Length of practice as a dentist was categorized as <6, 6–10, 11–15, and >15 years. Number of patients treated per day was separated into the four categories of <11, 11–20, 21–30 and >30.

Exposure to NSIs in the survey questionnaire was defined as being injured by a needlestick and sharps more than once per week within the last two weeks prior to survey. Because there are more than one dentist in a hospital and most clinics only have a single dentist in Taiwan, we sent at least four questionnaires to selected hospitals and only one questionnaire to selected clinics. The fact that the survey was specifically sent to dentists in the selected hospitals and clinics was addressed with the questionnaire. An honorarium of nearly US$3 was attached with the questionnaire in order to increase the response rate. After 2 weeks, we mailed the questionnaires again and called the selected facilities to ask the dentists to return the completed questionnaires.

### Ethical statement

Written informed consent was obtained from the study participants, and the survey procedures were reviewed and approved by the institutional review board of Taipei Medical University Hospital. (Approval number: TMUH-04-11-02).

### Statistical analysis

We used frequencies and percentages to describe the characteristics of dentists and facilities. We performed the correlation analysis to test whether compliance behaviors were correlated with each other. The odds ratio (OR) and 95% confidence interval (CI) were estimated from univariate logistic regression analyses to determine the association between risk factors and exposure to NSIs. We further conducted multivariable logistic regression models adjusted for age, years of practice, and number of patients treated, which were recognized as significant covariates in the univariate analysis. We conducted analyses using the statistical software package SPSS, vers. 17 (SPSS, Chicago, IL, USA). We considered *P* value of <0.05 to be statistically significant.

**Figure 1:**
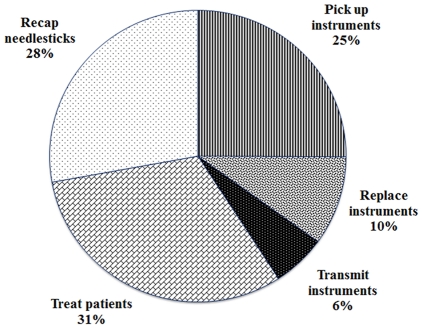
Self-reported behaviors associated with exposure to more than one NSI per week among dentists at hospitals and clinics in Taiwan in 2010 (*n* = 100).

## Results

In total, 434 of 580 dentists returned the survey questionnaires, for an overall response rate of 74.8%. The response rate was 77.9 percent (187 of 240) in hospitals, which was similar to 72.6 percent (247 of 340) in clinics (*P* = 0.15). An overview of study participants is given in Table 1. Among the participants, 317 (74.6%) were male. Half of the dentists were under 40 years old, and 191 of 434 (44.8%) had more than 15 years of experience in dentistry. About 46.7% of participants treated 11∼20 patients per day, and 2.6% participants treated more than 30 patients per day. We observed that 37.7% of healthcare facilities accredited by the Taiwan Joint Commission on Hospital Accreditation provide a postgraduate year (PGY) for dentists. Approximately 43.1% of dentists worked in hospitals, and most of them (83.0%) received information on infection control from journals, textbooks, seminars, and conferences.

**Table 2:** Logistic Regression Analyses of Demographic Factors Associated with NSIs.

Variable	Dentists with NSIs	Dentists without NSIs	Univariate odds ratio	Adjusted odds ratio
	(*n* = 100)	(*n* = 334)	(95% CI)	(95% CI)
Gender				
Male	74 (77.1)	243 (73.9)	Reference	Reference
Female	22 (22.9)	86 (26.1)	1.19 (0.69–2.03)	1.07 (0.59–1.93)
Age (years)*				
<31	14 (14.3)	97 (29.0)	Reference	Reference
31∼40	20 (20.4)	85 (25.4)	1.63 (0.77–3.42)	1.91 (0.68–5.42)
41∼50	40 (40.8)	87 (26.0)	3.18 (1.62–6.25)	4.61 (0.95–22.46)
>50	24 (24.5)	65 (19.5)	2.55 (1.23–5.30)	3.82 (0.72–20.30)
Length of practice as a dentist (years)*				
<6	17 (17.5)	103 (31.3)	Reference	Reference
6∼10	15 (15.5)	55 (16.7)	1.65 (0.76–3.56)	0.97 (0.34–2.79)
11∼15	8 (8.2)	37 (11.2)	1.31 (0.52–3.28)	0.46 (0.11–1.91)
>15	57 (58.8)	134 (40.7)	2.57 (1.41–4.69)	0.71 (0.15–3.41)
Number of patients treated per day				
<11	31 (31.3)	133 (40.2)	Reference	Reference
11∼20	50 (50.5)	151 (45.6)	1.42 (0.85–2.35)	1.15 (0.65–2.03)
21∼30	13 (13.1)	41 (12.4)	1.36 (0.65–2.84)	1.12 (0.50–2.47)
>30	5 (5.1)	6 (1.8)	3.57 (1.02–12.47)	2.58 (0.70–9.44)
Information source for dental infection control				
Newspaper or internet	18 (18.0)	56 (16.8)	Reference	Reference
Journals or textbooks	39 (39.0)	144 (43.1)	0.84 (0.44–1.59)	0.79 (0.39–1.56)
Seminars or conferences	43 (43.0)	134 (40.1)	0.99 (0.53–1.87)	0.80 (0.40–1.59)
Practicing in a setting that provides a postgraduate year for dentists				
Yes	28 (29.5)	126 (40.1)	Reference	Reference
No	67 (70.5)	188 (59.9)	1.60 (0.97–2.63)	1.30 (0.70–2.43)
Level of practice setting				
Hospital	33 (33.0)	154 (46.1)	Reference	Reference
Clinic	67 (67.0)	180 (53.9)	1.73 (1.08–2.77)	1.35 (0.76–2.40)

NSIs indicates needlestick injuries;

CI, confidence interval. *: P < 0.05 for trend test.

Among the 434 participants, 100 (23.0%) reported that they had experienced more than one NSI per week. The most common site of injury reported to be associated with NSIs was the fingers at 90.8%. Behaviors reported to be associated with NSIs are displayed in Figure 1. The most common NSIs occur when treating patients (31%), recapping a needle (28%), and picking up instruments (25%). Replacing instruments (10%) and transmitting instruments (6%) also accounted for smaller proportions of NSIs.

A comparison of dentists with and those without NSIs showed significant differences in age, years of practice, and level of the healthcare facility (Table 2). Dentists with NSIs were about 3.18-times (95% confidence interval (CI), 1.62–6.25) more likely to be aged 41∼50 years, 2.57-times (95% CI, 1.41–4.69) more likely to have practiced for more than 15 years, 3.57-times (95% CI, 1.02–12.47) more likely to treat over 30 patients per day, and 1.73-times (95% CI, 1.08–2.77) more likely to work in a clinic. However, adjusting for confounders changed the ORs, making these variables non-significant, such as age, length of practice as a dentist, number of patients treated per day and clinic setting. In terms of gender, healthcare facilities providing PGY training courses, and information sources about infection control, no significant differences were found.

**Table 3:** Logistic Regression Analyses of Infection-Control Procedures Associated with NSIs.

Variable	Dentists with NSIs	Dentists without NSIs	Univariate odds ratio	Adjusted odds ratio
	(*n* = 100)	(*n* = 334)	(95% CI)	(95% CI)
Compliance to all infection-control procedures				
Yes	31 (31.0)	141 (42.2)	Reference	Reference
No	69 (69.0)	193 (57.8)	1.63 (1.01–2.62)	1.44 (0.87–2.39)
Wearing gloves				
Yes	91 (91.0)	317 (94.9)	Reference	Reference
No	9 (9.0)	17 (5.1)	1.84 (0.79–4.27)	1.40 (0.56–3.51)
Wearing a protective uniform				
Yes	94 (94.0)	321 (96.1)	Reference	Reference
No	6 (6.0)	13 (3.9)	1.57 (0.58–4.26)	1.09 (0.36–3.26)
Sterilization of general dental examination instruments				
Yes	93 (93.0)	324 (97.0)	Reference	Reference
No	7 (7.0)	10 (3.0)	2.43 (0.90–6.58)	3.01 (1.05–8.60)
Sterilization of extraction instruments				
Yes	97 (97.0)	328 (98.2)	Reference	Reference
No	3 (3.0)	6 (1.8)	1.69 (0.41–6.88)	1.68 (0.39–7.18)
Sterilization of handpieces				
Yes	83 (83.0)	294 (88.1)	Reference	Reference
No	17 (17.0)	40 (12.0)	1.50 (0.81–2.79)	1.34 (0.70–2.59)
Sterilization of burs				
Yes	67 (67.0)	238 (71.3)	Reference	Reference
No	33 (33.0)	96 (28.7)	1.22 (0.75–1.97)	1.18 (0.71–1.96)
Periodic monitoring of sterilizers				
Yes	57 (57.0)	221 (66.2)	Reference	Reference
No	43 (43.0)	113 (33.8)	1.47 (0.93–2.32)	1.24 (0.76–2.03)
Periodic disinfection of waterline				
Yes	77 (77.0)	287 (85.9)	Reference	Reference
No	23 (23.0)	47 (14.1)	1.82 (1.04–3.18)	1.71 (0.93–3.16)

NSIs indicates needlestick injuries;

CI, confidence interval.

The association between compliance to infection-control procedures and exposure to NSIs was shown in Table 3. Compliance in wearing gloves was positively correlated with compliance in wearing a protective uniform. (P<0.05) According to a logistic regression analysis, dentists who were not compliance to all infection-control procedures, were found to be 1.63-times (95% CI, 1.01–2.62) more likely to experience an NSI. Dentists who did not carry out periodic disinfection of the waterline were less compliant and were 1.82-times (95% CI, 1.04–3.18) more likely to experience an NSI. However, adjusting for confounders changed the ORs, making these variables non-significant, such as not compliant with all infection-control procedures, and periodic disinfection of waterline. After adjustment for age, years of practice, and number of patients treated, dentists not compliant to sterilize general dental examination instruments were at an increased risk for NSIs (adjusted OR, 3.01, 95% CI, 1.05–8.60).

**Table 4:** Logistic Regression Analyses of Knowledge and Attitudes Related to Blood-borne Pathogens Associated with NSIs.

Variable	Dentists with NSIs	Dentists without NSIs	Univariate odds ratio	Adjusted odds ratio
	(*n* = 100)	(*n* = 334)	(95% CI)	(95% CI)
Path of HBV transmission				
Correct	35 (36.5)	77 (24.8)	Reference	Reference
Incorrect	61 (63.5)	233 (75.2)	0.57 (0.35–0.93)	0.55 (0.33–0.92)
Attitudes toward caring for HBV carriers				
Care for them	97 (99.0)	316 (95.5)	Reference	Reference
Refer them to others	1 (1.0)	15 (4.5)	0.21 (0.02–1.66)	0.19 (0.02–1.51)
Worried of being infected with HBV				
No	20 (20.4)	106 (31.8)	Reference	Reference
Yes	78 (79.6)	227 (68.2)	1.82 (1.05–3.13)	1.81 (1.03–3.21)
Oral signs of HIV				
Correct	51 (54.8)	211 (67.0)	Reference	Reference
Incorrect	42 (45.2)	104 (33.0)	1.67 (1.04–2.67)	1.38 (0.83–2.30)
Attitudes about caring for HIV carriers				
Care for them	48 (51.6)	156 (49.8)	Reference	Reference
Refer them to others	45 (48.4)	157 (50.2)	0.93 (0.58–1.48)	0.77 (0.46–1.27)
Worried about being infected with HBV				
No	12 (12.0)	63 (18.9)	Reference	Reference
Yes	88 (88.0)	270 (81.1)	1.71 (0.88–3.31)	1.72 (0.87–3.40)

NSIs indicates needlestick injuries;

CI, confidence interval;

HBV, hepatitis B virus;

HIV, human immunodeficiency virus.

Table 4 shows the knowledge and attitudes related to HBV and HIV associated with NSIs. In the logistic regression models, dentists with NSIs were 1.82-times (95% CI, 1.05–3.13) more worried about being infected by HBV and the result remained significant after adjustment for age, years of practice, and number of patients treated. Dentists with NSIs were 1.67-times (95% CI, 1.04–2.67) more likely to have incorrect knowledge of oral signs of HIV, compared to dentists without NSIs. Incorrect knowledge of HBV transmission pathways has a significantly negative association with NSIs (adjusted OR, 0.55, 95% CI, 0.33–0.92).

## Discussion

This is the first nationwide study in Taiwan among dentists to address important aspects of the epidemiology of and factors contributing to NSIs. Our findings indicate that dentists are at high risk for NSIs, but the overall awareness of infection-control procedures is insufficient. Only a limited number of studies on the occupational hazards of dentists have been published to date, and those only described the frequency of NSIs [17], [24], [25]. Additionally, most published studies analyzed NSI exposures of all healthcare workers, and it is difficult to identify the actual risk of NSIs for dentists [16], [28]–[30]. Furthermore, no studies analyzed related risk factors of NSIs for dentists. The present study clearly demonstrates the need for increased awareness of the risks of occupational exposure to NSIs among dentists in Taiwan.

In our study, 100 (23.0%) of 434 dentists reported that they had experienced more than one NSI per week. The rate of NSIs detected here was impressive and higher than rates reported among dentists in the US (20.8%) [31]. This estimated rate seemed to increase with the length of the study period for exposure to NSIs. For example, in a Brazilian study, 31.1% of dentists reported a percutaneous exposure in the previous year [32]. In a Danish study, 54.3% of dentists reported at least one NSI during their profession career [17]. In another Romanian study, 87% of dentists reported a percutaneous injury in the previous year [33]. However, all of those studies were conducted in only a single city.

As reported by previous studies, our study confirms that fingers are the most common site of injury [31], [34], [35]. In agreement with findings reported by others [2], [15], [16], [25], [36], we observed that behaviors reported most frequently related to NSIs were treating patients (31%) and recapping a needle (28%). We also found that NSIs need to be considered when picking up an instrument because it accounts for as much as 25% of NSIs. Dentists should be more cautious when treating patients and also using instruments.

Dentists with NSIs were found to be less compliant with infection-control procedures. This highlights the risk for transmission of blood-borne pathogens. Our finding confirms a previous report of the association between compliance with infection-control procedures and fewer NSIs. In addition, dentists who reported experience with NSIs were more likely to have incorrect knowledge of HIV and worry of being infected by HBV. A previously published report also found a correlation between negative concerns regarding the treatment of HIV patients and greater NSI exposures [37]. Dentists in clinics were more likely to be exposed to NSIs, suggesting that more efforts need to be made to ensure that clinics are as safe as hospitals.

Our data showed that younger dentists had fewer NSIs than older ones, and more-experienced dentists reported more NSIs. Similarly, several studies demonstrated that more-experienced dentists were less compliant with infection-control procedures and were more easily exposed to NSIs [37], [38]. However, another previous report suggested that professional experience was not associated with occupational NSI exposure, but an overload of activities was [32]. Another interesting finding in our study was that dentists treating more than 30 patients per day were at a 3.57-fold higher risk of NSIs compared to dentists treating fewer than 10 patients per day. This highlights the issue of fatigue and its relation to an increased risk of NSIs.

The negative association between incorrect knowledge of path to HBV transmission and exposure to NSIs contradicted our hypothesis. The reason is unclear, but may be due to reverse causation. Dentists used to be exposed to NSIs tend to look for more knowledge about path to HBV transmission after NSIs in order to prevent to be infected. This may also apply to the variable “Worried about being infected”.

The significant effects of age, length of practice, number of patients treated per day and level of practice setting in the univariate model became insignificant when adjusted with each other. This could be caused by the fact that these factors were highly associated with each other, and accounted for the similar measures–cumulative exposure to practice. Although the correlation between each other is not as severe as collinearity which may distort the validity of estimated results, considering all demographic features in our study is possibly not sufficiently powered. Interestingly, we found that length of practice becomes protective, although insignificant, in multivariable analysis. This indicated that identifying as a risk factor of NSI in the univariate analysis is likely to be confounded by age. Dentists practicing longer are more likely to be older. After controlling for age and other factors, longer length of practice tend to be protective to NSI.

Similarly, the positive associations for not compliant with all infection-control procedures and periodic disinfection of waterline in the univariate analysis become insignificant in multivariable analysis with adjustment of age, years of practice, and number of patients treated. This may be also caused by underpower. A larger scale of study is therefore needed in the future.

Three study limitations should be noted. The primary limitation of the study is the cross sectional design which does not allow for assessment of temporal associations. For example, issues with reverse causality may exist such that opinions have been influenced by the NSI. Second, given that exposure to NSIs is based on self-reported data, it is likely that not all NSIs were reported in the survey. A prior study found that nearly four in five NSIs were not reported in Taiwan [39]. Studies from other countries also showed that 22%∼78% of NSIs go unreported [40], [41]. Our study therefore likely underestimated the true risk of NSIs. Third, our analysis of compliance with infection-control procedures was also based on self-reported data, which could have resulted in an overestimation of compliance.

Our study revealed that the risk of occupational NSIs is similarly heightened by an older age, more years of practice, working in clinics, lower compliance with infection-control procedures, having insufficient knowledge of blood-borne pathogens, and being more worried about being infected by blood-borne pathogens. High rates of NSIs and low compliance with infection-control procedures highly contributed to the chance of getting a blood-borne pathogen infection. This study may help in designing prevention strategies for NSIs. Further intensive educational interventions to increase their awareness and tighter infection-control procedures are suggested for dentists, particularly those who are older, have more years of practice, and work in clinics. It is important that recommended infection-control guidelines and knowledge of blood-borne pathogens be repeatedly emphasized throughout continuing dental education. Regardless, prevention of occupational exposure to NSIs should be a priority as a national policy. All dental practices should provide an educational project for training staff members to prevent NSIs and should have a comprehensive written program including procedures for reporting and providing medical follow-up for NSIs.
